# Zollinger Ellison Syndrome in a Patient with Multiple Endocrine Neoplasia Type 1: A Classic Presentation

**DOI:** 10.1155/2019/9605769

**Published:** 2019-06-03

**Authors:** Ishani Shah, Neil Vyas, Kambiz S. Kadkhodayan

**Affiliations:** ^1^Department of Internal Medicine, Creighton University St. Joseph's Hospital and Medical Center, Phoenix, AZ, USA; ^2^Department of Gastroenterology, Creighton University St. Joseph's Hospital and Medical Center, Phoenix, AZ, USA

## Abstract

Zollinger Ellison Syndrome (ZES) is characterized by a wide spectrum of conditions including severe gastroesophageal reflux disease, peptic ulcer disease, watery diarrhea, and weight loss. We present a case of a 60-year-old woman being evaluated for severe dyspepsia, vomiting, and chronic diarrhea, who was diagnosed to have ZES associated with a pancreatic neuroendocrine tumor, in the setting of multiple endocrine neoplasia (MEN) type 1. Although cases of ZES have been reported previously, we believe that our case is a classic presentation of ZES diagnosed on the basis of typical radiologic, endoscopic, and endosonographic features.

## 1. Introduction

Zollinger Ellison Syndrome (ZES) is caused by ectopic hypersecretion of gastrin from pancreatic or duodenal neuroendocrine tumors (NETs), commonly referred to as gastrinomas. Such high gastrin levels cause gastric acid overproduction, leading to a typical presentation, usually consisting of peptic ulcer disease and severe diarrhea. We present a classic case of ZES in a patient with multiple endocrine neoplasia (MEN) type 1.

## 2. Case Presentation

A 60-year-old woman presented to our hospital with severe nausea, vomiting, watery diarrhea, and burning epigastric pain for a duration of one week. Her epigastric pain was associated with severe acid reflux, which had been intermittently present for a duration of two years and was resistant to over-the-counter low-dose proton pump inhibitor (PPI) therapy. Her past medical history was negative for any evidence of gastrointestinal (GI) bleed. Interestingly, the patient had a daughter who had been diagnosed with multiple endocrine neoplasia (MEN) type 1 a year prior to presentation. On physical exam, she was afebrile with stable hemodynamics. Abdominal palpation revealed mild epigastric tenderness without any guarding or rigidity. Cardiopulmonary exam was within normal limits.

**Figure 1:**
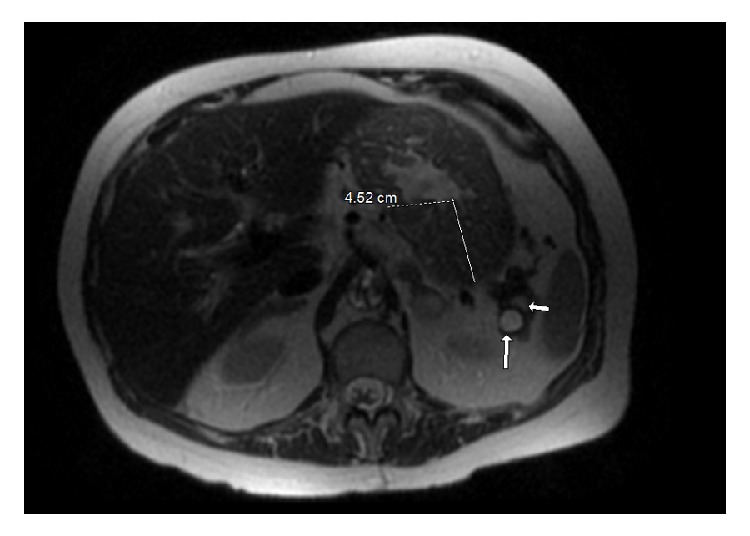
MRI abdomen showing diffuse gastric wall thickening (4.52 cm) with a small pancreatic cystic lesion (arrows).

Significant laboratory findings included WBC count of 15,000/microL, potassium of 3 mmol/L, magnesium of 0.7 mg/dL, and calcium of 11.8 mg/dL. Lipase level was within normal limits. Other pertinent laboratory values included fasting serum gastrin level of 1603 pg/mL (0-180 pg/mL), chromogranin A level of 14600 ng/mL (0-100 ng/mL), prolactin hormone level of 21 ng/mL (2-29 ng/mL), and parathyroid hormone (PTH) level of 473 pg/mL (10-65 pg/mL). She did not have any history of prior gastric surgeries, gastroparesis, or renal disease, to possibly explain her elevated gastrin level. An infectious workup for her diarrhea, including* Clostridium difficile* toxin and a stool PCR panel for common enteric pathogens, was negative. Subsequently, an extensive workup for evaluation of MEN was done, which revealed a unilateral parathyroid adenoma on neck imaging and diffuse stomach wall thickening along with pancreatic cystic lesions in body (1.2 cm) and tail (0.7 cm) on abdominal MRI (Figure 1). Testing for pituitary disease was negative.

**Figure 2:**
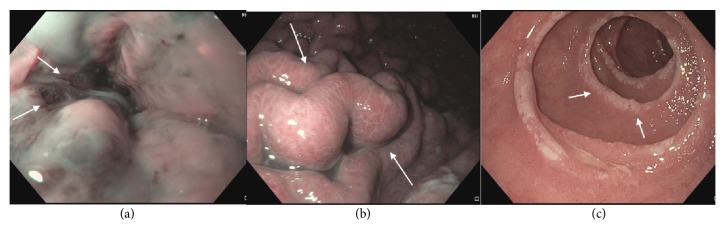
EGD showing LA grade D esophagitis in the distal esophagus (a), hypertrophic rugae in the gastric body (b), and multiple postbulbar ulcers in third part of the duodenum (c), as indicated by arrows.

**Figure 3:**
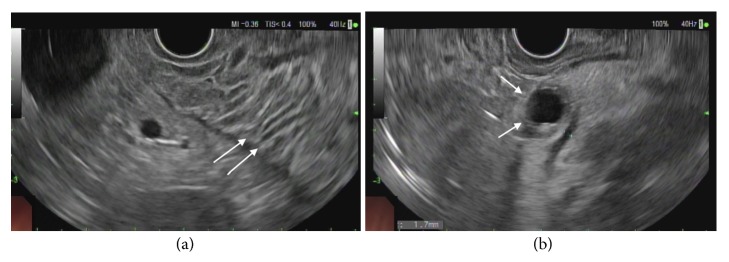
EUS showing hypertrophic gastric rugae (a) and neuroendocrine tumor in the pancreatic neck (b), respectively, as indicated by arrows.

**Figure 4:**
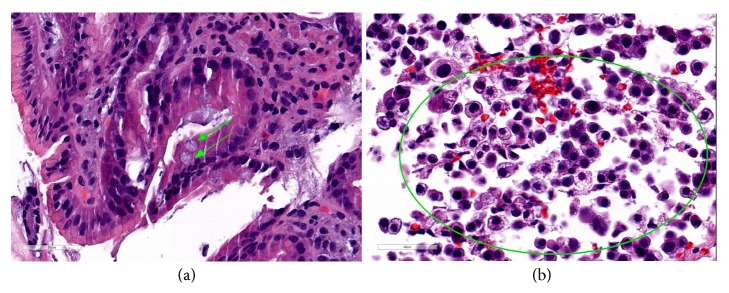
Gastric biopsy (a) showing patchy hypertrophic gastritis and intestinal metaplasia (green arrows) while pancreatic aspirate (b) showing neuroendocrine cells (area enclosed within circle).

An esophagogastroduodenoscopy (EGD) was performed for further evaluation of her symptoms, which revealed severe reflux esophagitis, diffusely hypertrophic gastric rugae and multiple postbulbar ulcers in the duodenum (Figures 2(a), 2(b), and 2(c)). Endoscopic ultrasound (EUS) subsequently revealed diffuse thickening of the gastric rugae, predominantly of echo-layers I-III (Figure 3(a)). In addition, the patient was found to have a cystic lesion in the pancreatic neck with thick hypoechoic walls (Figure 3(b)). Random biopsies of the gastric antrum and body revealed patchy chronic gastritis with intestinal metaplasia (Figure 4(a)) while FNA from pancreatic cyst revealed well differentiated NET (Figure 4(b)).

The patient eventually underwent a distal pancreatectomy and parathyroidectomy with clinical improvement. The remainder of her hospital course was uncomplicated and she was discharged home on high-dose PPI and octreotide.

## 3. Discussion

Patients presenting with gastroesophageal reflux disease (GERD) unresponsive to standard PPI therapy and chronic diarrhea should be evaluated for ZES from gastrin producing NETs, also known as gastrinomas. Gastrinomas leading to ZES are predominantly duodenal; about 25% are pancreatic in origin [1]. The annual incidence of gastrinomas is about 4 to 5 per million population [2]. Most ZES patients present with symptoms of acid reflux (52%-56%) and peptic ulcer disease (73%-98%), while other common symptoms include chronic diarrhea (60%-75%) and weight loss (7%-53%) [3, 4]. Fasting serum gastrin (FSG) levels of more than 10 times the upper limit of normal along with a gastric pH of less than 2 are highly predictive of ZES. However, there is data to suggest that FSG levels can be elevated in various other conditions such as antral gastric cell hyperplasia,* H. Pylori *infection, acute renal failure, gastric outlet obstruction, and short bowel syndrome [5]. Additionally, secretin stimulation testing is commonly used for distinguishing ZES from other causes of hypergastrinemia such as antral G-cell hyperplasia and gastric outlet obstruction. An increase in gastrin level of less than ten times upper limit of normal or gastric pH of <2 essentially indicates an alternate diagnosis. It works on the principle that secretin stimulates gastrin secretion from gastrinoma cells but inhibits normal gastric G cells.

While clinical and laboratory findings can usually be diagnostic of ZES, radiologic and endoscopic investigations are required for localizing gastrinomas and evaluating for metastases. CT, MRI, and somatostatin receptor scintigraphy are often useful; however definitive diagnosis is frequently established via endoscopic evaluation as seen in our case. Similar to our case, upper endoscopy in most cases of ZES shows reflux esophagitis, hypertrophic gastric folds, and multiple ulcers scattered over first (75%) and third (14%) parts of the duodenum and sometimes over the jejunum (11%) [6]. Additionally, EUS is an extremely sensitive (93%) and specific (95%) test for diagnosing pancreatic NETs and has been shown to be superior to CT, MRI, and other invasive diagnostic measures such as angiography and secretin stimulation testing [7–10]. It can detect pancreatic lesions as small as 2 or 3 mm in diameter and provides the opportunity to perform FNA of suspicious lesions.

Around 80% of gastrinomas are sporadic; however 20% are associated with MEN type 1, an autosomal dominant condition associated with predisposition to tumors in the hyperparathyroidism glands, pancreas, and pituitary [11]. About 40% of patients with MEN type 1 initially present with symptoms of ZES [12]. Therefore, an extensive workup for MEN should be carried out in all patients with ZES. In many studies, EUS has consistently been proven to be superior to other imaging modalities for diagnosing pancreatic NETs in patients with a suspected or established diagnosis of MEN type 1 [8, 13, 14].

In conclusion, our case is a classic blend of clinical, radiologic, endoscopic, and endosonographic features of ZES in a patient with MEN type 1. It highlights the importance of early recognition of these findings for prompt diagnosis and appropriate treatment.
